# Single-Voxel MR Spectroscopy of Gliomas with s-LASER at 7T

**DOI:** 10.3390/diagnostics13101805

**Published:** 2023-05-19

**Authors:** Martin Prener, Giske Opheim, Zahra Shams, Christian Baastrup Søndergaard, Ulrich Lindberg, Henrik B. W. Larsson, Morten Ziebell, Vibeke Andrée Larsen, Mark Bitsch Vestergaard, Olaf B. Paulson

**Affiliations:** 1 Neurobiology Research Unit, Department of Neurology, Rigshospitalet Blegdamsvej, 2100 Copenhagen, Denmark; martin.prener@nru.dk (M.P.); gopheim@gmail.com (G.O.); 2 Department of Radiology, Rigshospitalet Blegdamsvej, 2100 Copenhagen, Denmark; vibeke.andree.larsen@regionh.dk; 3 Center for Image Sciences, University Medical Centre Utrecht, Heidelberglaan 100, 3508 GA Utrecht, The Netherlands; z.shams@umcutrecht.nl; 4 Department of Neurosurgery, Rigshospitalet Blegdamsvej, 2100 Copenhagen, Denmark; christian.baastrup.soendergaard.01@regionh.dk (C.B.S.); mzi@regionsjaelland.dk (M.Z.); 5 Functional Imaging Unit, Department of Clinical Physiology, Nuclear Medicine and PET, Rigshospitalet Glostrup, 2600 Copenhagen, Denmark; ulrich.lindberg@regionh.dk (U.L.); henrik.bo.wiberg.larsson@regionh.dk (H.B.W.L.); mark.bitsch.vestergaard@regionh.dk (M.B.V.); 6 Department of Clinical Medicine, University of Copenhagen, 2200 Copenhagen, Denmark

**Keywords:** gliomas, ultra-high field MR, 7-Tesla MRS, MR spectroscopy, glioma biomarkers

## Abstract

Background and Purpose: Magnetic resonance spectroscopy (MRS)—a method of analysing metabolites in vivo—has been utilized in several studies of brain glioma biomarkers at lower field strengths. At ultra-high field strengths, MRS provides an improved signal-to-noise-ratio and spectral resolution, but 7T studies on patients with gliomas are sparse. The purpose of this exploratory study was to evaluate the potential clinical implication of the use of single-voxel MRS at 7T to assess metabolic information on lesions in a pilot cohort of patients with grade II and III gliomas. Methods: We scanned seven patients and seven healthy controls using the semi-localization by adiabatic-selective refocusing sequence on a Philips Achieva 7T system with a standard dual-transmit head coil. The metabolic ratios were calculated relative to water and total creatine. Additionally, 2-hydroxyglutarate (2-HG) MRS was carried out in four of the patients, and the 2-HG concentration was calculated relative to water. Results: When comparing the tumour data to control regions in both patients and healthy controls, we found that the choline/creatine and myo-inositol/creatine ratios were significantly increased and that the N-acetylaspartate/creatine and the neurotransmitter glutamate/creatine ratios were significantly decreased. The N-acetylaspartate/water and glutamate/water ratios were also significantly decreased. The lactate/water and lactate/creatine ratios showed increases, although not significant. The GABA/water ratio was significantly decreased, but the GABA/creatine ratio was not. MRS spectra showed the presence of 2-HG in three of the four patients studied. Three of the patients, including the MRS 2-HG-negative patient, were operated on, and all of them had the IDH mutation. Conclusion: Our findings were consistent with the existing literature on 3T and 7T MRS.

## 1. Introduction

Gliomas are tumours originating from the brain’s glial cells. Using classical histological analyses, gliomas can be differentiated into low-grade and high-grade gliomas through the following features: atypia, anaplasia, mitotic activity, microvascular proliferation, and the presence of necrosis. The different subtypes differ substantially in overall survival, from 16 years for low-grade oligodendrogliomas all the way down to 18 months for malignant gliomas [1,2]. Since 2016, the WHO has included molecular parameters in the assessment, and in 2021, the parameters were further updated to include more molecular markers, e.g., IDH mutation, into the classification of gliomas, which are also obtained through histological examination of tumour tissue [3,4].

To classify a tumour, an invasive procedure such as a biopsy or tumour resection is required. Magnetic resonance spectroscopy (MRS) is a non-invasive method of analysing the metabolites of living tissue. Several of these metabolites have interest for the characterization of brain tumours. For example, N-acetylaspartate (NAA) is a biomarker that is associated with healthy, functioning brain tissue. It is synthesized in the neurons and is decreased in gliomas [5,6]. Choline (Cho) is a marker of the breakdown of phospholipids of the membrane. It is associated with increased cell proliferation/membrane turnover, and is thus elevated in tumour cells [7]. Total creatine (tCr) is involved in energy metabolism [8]. Its concentration is typically lower in tumour cells [9]. These compounds and their ratios represent important parameters in the MRS evaluation of brain tumours. Lactate is the final product of anaerobic glycolysis and depends on the level of necrosis in the gliomas. Lactate/tCr might be increased in gliomas [10]. Lactate may also be increased in tumour tissue despite adequate oxygen supply as a consequence of tumour growth, also known as the so-called Warburg effect. The concentration of lactate is low in healthy brain cells and is just visible in the spectra at rest [11]. Myo-inositol (mIns) is part of the second messenger system, and its concentration is higher in glial cells than in neurons [12]. Gamma-aminobutyric acid (GABA) is the primary inhibitory neurotransmitter in the brain which, due to its low concentration, is not detectable with traditional MRS methods at a conventional field strength of 3 Tesla [13]. Glutamate (Glu) and glutamine (Gln) are detectable at 3T, but it is not possible to separate the two [13]. Glutamate is the primary excitatory neurotransmitter in the human brain [14], and it is regulated through a glutamine–glutamate and tricarboxylic acid cycle (TCA) cycle. Glutamine is known to stimulate the growth of gliomas [15]. Glutamate may be reduced as consequence of a reduced number of neurons and may also be influenced by an enabled glutamine catabolism as part of the Warburg effect [16].

Mutations in the isocitrate dehydrogenase (IDH1/2) enzyme, which is typically found in grade II and III gliomas, result in the accumulation of 2-hydroxyglutarate (2-HG) in tumour cells [17]. IDH-mutated gliomas have shown improved survival rates compared to the IDH wild-type tumours. On the other hand, the downstream effects of increased levels of 2-HG in IDH-mutated gliomas may alter global cellular metabolism, leading to the malignant transformation of tumour cells [18]. Therefore, 2-HG can be an important biomarker for diagnosing and monitoring therapy responses in IDH-mutated gliomas. This metabolite can be detected noninvasively by MR spectroscopy at a high field strength [19,20,21]. In the 2021 WHO tumour classification, the presence of the IDH mutation is what separates astrocytomas from glioblastomas, further emphasizing the clinical utility of 2-HG-MRS [4].

Although extensive information on the concentration and presence of the above-mentioned metabolites in brain tumours can be obtained via MRS, these findings can still only be considered supplements and not replacements for histology [22]. However, equally as important is that the non-invasive, in vivo nature of MRS allows for the evolution in a single patient to be followed and hopefully also for the recurrent tumour to be distinguished from radiation-induced brain lesions.

Compared to clinical field strengths of 1.5T and 3T, 7T MR has a higher signal-to-noise ratio (SNR) [23]. Due to the increased SNR and metabolite spectra separation, MRS at 7T allows for the separation of Glu and Gln [13]. However, the literature using single-voxel MRS or MRS imaging of the conventional metabolites at 7T is still sparse, and more investigations into their use in specific patient populations are warranted.

In our study, we use single-voxel 7T MRS imaging of patients with gliomas to investigate classical glioma biomarkers that are well known from 3T MRS. We compare our results to those in larger studies that used 3T and to the results in the published 7T literature [24,25].

## 2. Methods

### 2.1. Participants

This project was approved by the Ethical Committee of the Capital Region of Denmark (H-17013561_6 for controls and H-4-2014-134_3 for patients), and all participants signed consent forms prior to scanning. The data processing was approved by the Danish Data inspectorate (RH-2016-387, I-Suite nr. 05172). Further, as the 7T MR scanner is not CE-marked (Conformité Européenne), the study was also approved by the Danish Medicines Agency (2016101738, revised 2017081122). The patients were recruited from the Department of Neurosurgery at Copenhagen University Hospital Rigshospitalet. Originally, fourteen patients with gliomas were referred; four were excluded during safety screening (three due to tattoos within the set safety margins of >30 cm from the coil and one due to pregnancy), two withdrew consent, and one was excluded as the scan was interrupted before its completion. Finally, we included seven healthy volunteers (3/4 females/males; age span, 22–69; median age, 31.7) and seven patients in the study, see Table 1. It should be further noted that none of the patients had received any treatment prior to the scans.

### 2.2. MR Acquisitions

This study was performed using an actively shielded Philips Achieva 7T MR system (Philips Healthcare, Best, the Netherlands) equipped with a two-channel volume transmit head coil with a 32-channel receiver array (Nova Medical Inc., Burlington, MA, USA). The scanner was installed at the Danish Research Centre for Magnetic Resonance, Copenhagen University Hospital Hvidovre. We used 3D based B_1_^+^ scaling, which greatly reduced the spatially varying inhomogeneities [26]. This improved tumour–tissue contrast, particularly in the temporal regions, which, in turn, provided a better basis for the placement of MRS voxels on 3D FLAIR images (see Section 2.3). On top of the B_1_^+^ scaling, we applied dielectric pads (size: 19 cm × 19 cm, Multiwave Technologies Ltd., Geneva, Switzerland) on both sides of the head to further minimize B_1_^+^-induced inhomogeneities. We also used interleaved volumetric fat-navigator-based prospective motion correction for the structural sequences [27], which reduced the need for re-scans and thus also patient discomfort caused by prolonged scan duration.

### 2.3. Structural Imaging (Used for Placement of MRS Voxels)

We acquired a 3D FLAIR sampled in the sagittal direction with the following parameters: repetition time (TR) = 7342 ms; echo time (TE) = 348 ms; inversion time (TI) = 2200 ms; acquisition voxel size = 0.7 mm × 0.7 mm × 1.4 mm; flip angle = 75 degrees; refocusing angle = 30 degrees; scan duration = 6 min. 44 s. In this study, the scan was used for MRS voxel placement, although it was also acquired as part of an additional structural imaging research protocol.

### 2.4. *^1^*H-MRS

**Figure 1:**
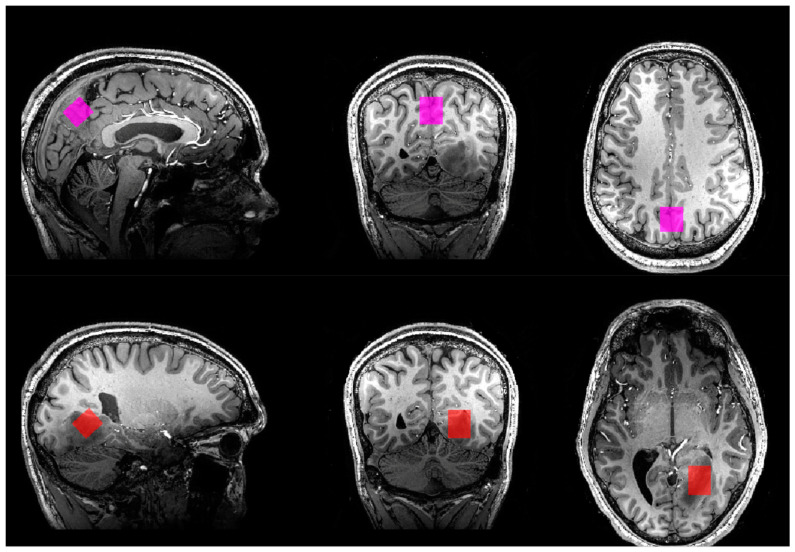
Voxel placement in all three planes in patient no. 1. (**Top**): praecuneus. (**Bottom**): tumour.

Semi-localization by adiabatic-selective refocusing (sLASER) sequences were used in the study [28]. A sLASER sequence for measuring the concentrations of glutamate, glutamine, creatine, phosphocreatine, total choline (tCho), *N*-acetylaspartate (NAA), *N*-acetylaspartyl glutamate (NAAG), GABA, lactate, and myo-inositol was acquired using the following sequence parameters: voxel size = 18.1 mm × 20.0 mm × 20.0 mm; TR = 4000 ms; TE = 32 ms; flip angle = 90 degrees; 32 transients; scan duration = 2 min. 8 s; 2048 complex data points; bandwidth = 4000 Hz. For patients, the voxels were placed in tumour regions (Figure 1). For both patients and control participants, a voxel of the same size as the tumour voxel was placed in the central praecuneus, covering both hemispheres in the parietal lobe (Figure 1).

In the patients, an additional sLASER sequence optimized for measuring 2-HG at TE = 110 ms [19,29] was used to acquire the spectrum from a voxel located in the tumour region. The following parameters were applied: voxel size = 18.1 mm × 20.0 mm × 20.0 mm; TR = 4000 ms; TE = 110 ms (TE1 = 11 ms, TE2 = 65 ms, and TE3 = 34 ms); flip angle = 90 degrees; 114 transients; scan duration = 7 min. 36 sec.; 2048 complex data points; bandwidth = 4000 Hz.

For both sLASER sequences, a second-order shim was applied. B_0_ was optimized in the volume of interest by FASTMAP [30], using shim terms up to the second order (second-order projection-based shimming, which is called pencil beam-second in Philips scanners). Variable power RF pulses with optimized relaxation delays (VAPOR) were used for water suppression [31]. An unsuppressed water signal was acquired via an additional single acquisition prior to the measurement of the water-suppressed data.

### 2.5. Spectral Fitting and Quantification

A visual inspection was performed to exclude spectra of very poor quality. LCModel (Version 6.3-1F) was used for the quantification of metabolite concentrations [32,33]. Basis sets for the short-echo-time sLASER sequence were generated using the Versatile Simulation, Pulses and Analysis (Vespa) software package [34]. The following metabolites were fitted in the model: alanine, aspartate, tCho, Cr, PCr, GABA, Gln, Glu, glycine, glutathione (GSH), mIns, lactate, NAA, NAAG, scyllo-inositol, taurine, phosphoethanolamine, and serine. The basis set also included macromolecules at chemical shifts of 0.9 ppm, 1.2 ppm, 1.7 ppm, and 2.0 ppm, and lipid signals at chemical shifts of 0.9 ppm, 1.3 ppm, and 2.0 ppm. The following concentrations were used for the further analyses: Glx (glutamate + glutamine), total creatine (tCreatine = creatine + phosphocreatine), Glu, Gln, tCho, total NAA (NAA + NAAG), GABA, lactate, and mIns. The spectra were fitted in the frequency span from 0.2 ppm to 4.0 ppm. For baseline flexibility, a default LCModel parameter was used.

For the analysis of the spectra acquired from the 2-HG optimized sLASER, LCModel basis sets were generated using VESPA. We performed the simulations with the same RF pulses and sub-TEs as those we used on the 7T Philips system. No volume localization was considered in the simulations. [29]. The following metabolites were fitted in the model: 2-HG, alanine, aspartate, tCho, Cr, PCr, GABA, Gln, Glu, glycine, GSH, mIns, lactate, NAA, NAAG, scyllo-inositol, and taurine.

For the sLASER with a long TE = 110 ms, no macromolecular basis functions were used. The DKNTMN parameter in LCModel was set to 0.2 so that the flexible spline baseline could compensate for the high variability of macromolecules and lipid signals [32]. Nevertheless, at TEs of longer than 100 ms, the macromolecules’ contribution can be neglected [35]. The unsuppressed water signal was used as a reference to obtain the absolute concentrations of the metabolites. The spectra were fitted in the frequency range of 4.2 ppm–0.2 ppm.

The signal-to-noise ratio (SNR) and linewidth (full width at half maximum; FWHM) from the LCModel analysis were used to assess spectral quality.

### 2.6. Analyses

Following the visual inspection of spectra and the assessment of the FWHM and SNR values, the data were analysed. The endpoints were the differences in the metabolite ratios in the brain tumour regions compared to the control regions: we compared metabolic ratios from the tumour to the praecuneus within each patient for all patients, and we additionally compared the metabolic ratios from the tumour to the praecuneus in the healthy controls, also for all patients. Lastly, we compared the metabolic ratios between the praecuneus from the patients to the healthy controls. We used the praecuneus as a reference region due to its low metabolic variance [36]. Four of the patients (1, 3, 4, and 6) had their tumour in a neighbouring region. Of note, we used both water and tCr values as references. tCr was used due to its relative stable concentration throughout the brain [37]. To evaluate the individual variation in normal tissue, we calculated the coefficient of variation (CV = SD/value) for the tCr and all metabolic ratios in the healthy controls.

For statistical comparison, we used a two-sided Wilcoxon rank-sum (Mann–Whitney) test with a 5% significance level. For each set of tests, we used a Bonferroni correction for multiple comparisons. With the comparison of 17 metabolic ratios twice, we Bonferroni-corrected our alpha threshold, 0.05/(17 × 2) = 0.00147.

The distance between the border of the tumour and the border of the praecuneus was estimated by an experienced neuroradiologist (VAL) and was based on primarily the 3D FLAIR sequence.

## 3. Results

**Figure 2:**
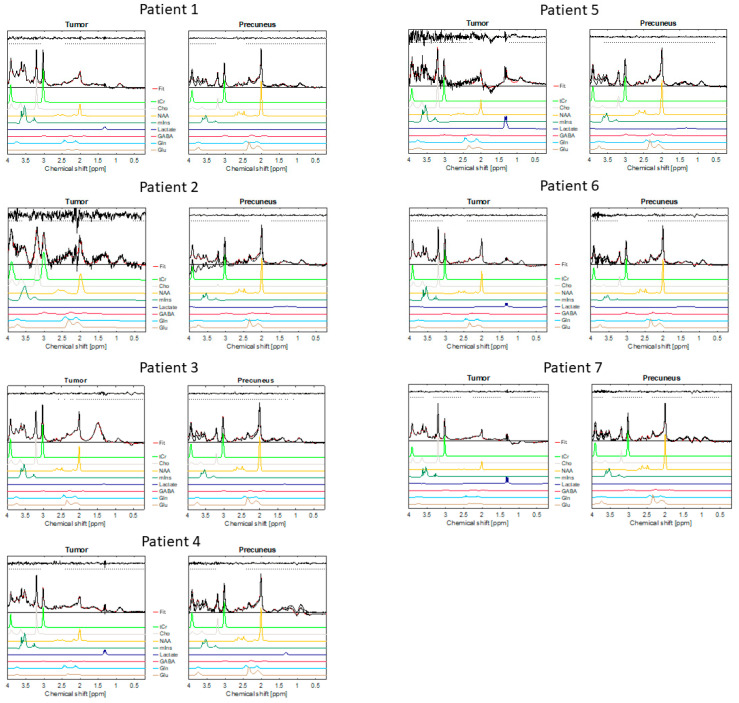
Shows the MRS spectra of all 7 patients. The red line in the spectra demonstrates the overall fit of all metabolites. The fits of the individual metabolites are likewise visualised by the coloured lines. The residuals of the overall fit are shown above the horizontal dotted grey line.

The metabolic ratios are shown in Table 2. Figure 2 shows the MRS spectra from the tumour and praecuneus for all seven patients.

### 3.1. Spectra Quality Assessment

As shown in Table 2 and Table 3, the FWHM values varied more in tumour regions (mean: 10.13 Hz; std: 7.15 Hz) than in the praecuneus regions in both patients (mean: 8.64 Hz; std: 1.49 Hz) and controls (mean: 7.75 Hz; std: 0.89 Hz). Following the consensus paper by Juchem et al., the overall quality of the spectra was good [38]. In addition, the SNR values were, on average, somewhat lower in tumour regions (mean: 24.4; std: 13.6) compared to the praecuneus in patients (mean: 38.3; std: 9.9) and controls (mean: 46.9; std: 5.46). The CRLB values were higher in the tumours for tCr, NAA, GABA, and glutamate compared to the praecuneus region in both patients and controls. This likely reflects the lower concentrations of these metabolites in the tumours making the fit of these metabolites more prone to noise. In contrast, the CRLB values for lactate were lower in the tumours than in the praecuneus, which is likely due to the higher lactate concentration in the tumours. The general decrease in SNR values and the increase in line width in tumours compared to control regions is expected from a large study at 1.5T [39], and it is known that the overall quality of tumour MRS spectra can be reduced [40,41].

### 3.2. Metabolite Variations in Healthy Controls

In healthy controls, the CV for the reference tCr was low, 8%, reflecting a stable reference substance with limited interindividual variation. For many of the metabolites, the CV was also low, indicating limited intraindividual variation. In general, all five of the nine variables with a concentration above 25% of the tCr concentration had coefficients of variation below 22%, with the lowest being 7%. The four metabolites with concentrations below 25% of the tCr had CVs ranging from 23 to 57%. The highest CV was observed for lactate with very low concentrations in normal brain tissue and with physiological variations.

### 3.3. Comparison of Metabolites between Tumour and Control Regions

As stated earlier we, used both water and tCr as reference substances. The tCr was not significantly decreased in tumour tissue compared to the praecuneus in patients, but it was significantly different compared to the praecuneus in healthy controls.

Two metabolites were significantly changed in tumour regions with both water and tCr used as references: NAA and Glu. NAA and NAA/tCr were significantly decreased in tumour tissue compared to the praecuneus in both patients and in healthy controls. Glu and Glu/tCr were also decreased in tumour tissue compared to the praecuneus in both patients and healthy controls.

GABA but not GABA/tCr was significantly decreased in tumour tissue compared to the praecuneus in both patients and healthy controls.

Cho/tCr and Cho/NAA were significantly increased in tumour tissue compared to the praecuneus in both patients and in healthy controls. The mIns/tCr ratio was significantly increased in tumour tissue compared to the praecuneus in patients and in healthy controls. Glu/Gln was significantly decreased in tumour tissue compared to the praecuneus in patients and healthy controls.

Of the non-significant changes in metabolic ratios, some showed a trend towards alterations. For Cho, though not significant when compared to the praecuneus in either group, concentrations revealed a trend towards an increase in tumour cells. As mentioned earlier, the GABA/tCr ratio was not significantly reduced. This was not significant, though it still shows a trend towards a decreased GABA/tCr ratio in tumour tissue. Lactate was increased but not significantly. The *p*-values were 0.01 and 4.1 × 10^−3^ in tumour tissue vs. the praecuneus in patients and the praecuneus in healthy controls, respectively.

The Gln/tCr ratio, Gln, mIns, and lipids did not show any significant differences in metabolic ratios in tumour tissues vs. the praecuneus both in patients and in healthy controls.

Between the control regions, the praecuneus in the patients and in the healthy controls, there were no differences in any of the metabolites.

### 3.4. 2-Hydroxyglutarate

**Figure 3:**
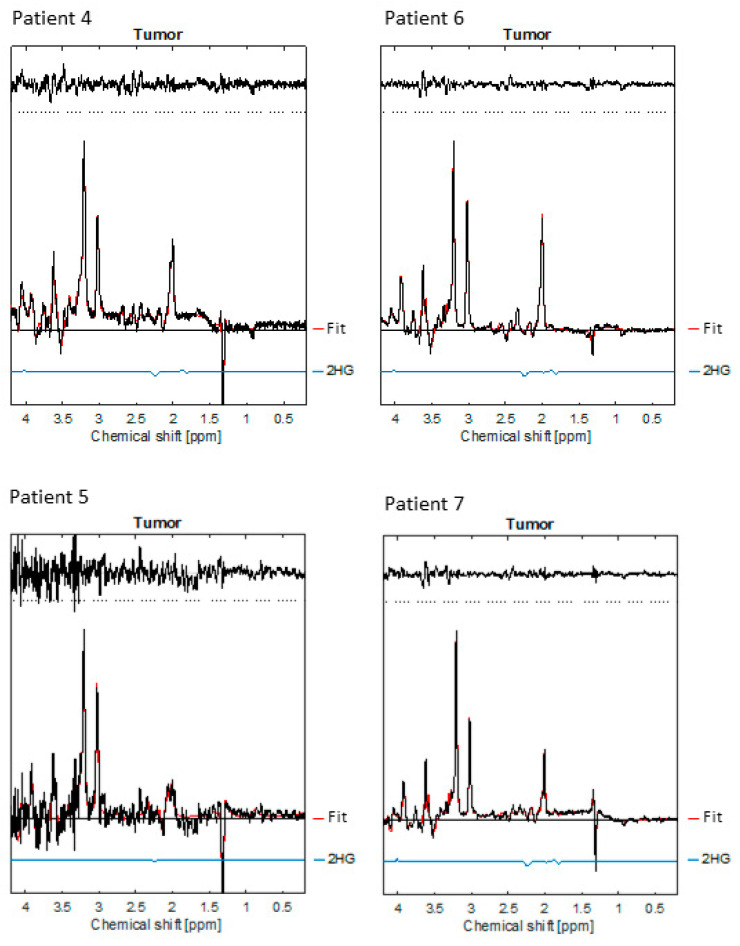
Shows the MRS spectra for the four patients scanned with the 2-HG sequence, pt. 4 to 7. See also Table 1.

Of the four patients scanned with the 2-HG sequence, three had the 2-HG producing IDH mutation histologically verified, see Figure 3. The respective concentrations of 2-HG can be seen in Table 1. As stated earlier, one of the patients is under watchful waiting and has not undergone surgery nor stereotactic biopsy, which means that histological analyses of their IDH status have not been possible. In the four patients, the 2-HG concentration varied between 0.144 mM (CRLB 199%) and 1.030 mM (CRLB 19%), see Table 1.

## 4. Discussion

### 4.1. Main Findings

The chemical composition of gliomas has previously been investigated using MRS at both conventional field strengths of 1.5 T or 3T and at an ultra-high field strength (7T). In Table 4, we have compared some of these findings.

### 4.2. Findings at 1.5T and 3T

Several studies have shown that decreased concentrations of NAA and increased concentrations of Cho, measured in reference to water or to other metabolites, are characteristic of low-grade gliomas (LGGs). Further, the mIns concentration was most often increased and the tCr concentration was decreased in LGGs. These metabolic patterns were observed at 1.5T as well as 3T MR imaging [10,42,43]. Bulik et al. (2013) further reviewed the literature on spectroscopic findings for gliomas from grade I to IV using 1.5T to 3T and confirmed these findings. Additionally, concentrations of NAA and Cho deviated in high-grade gliomas [44].

### 4.3. Findings at 7T

Li and co-workers found that Cho/NAA and Cho/tCr were significantly increased, but only for grade II gliomas [24]. This finding differs from those of Bulik et al., who found that the Cho increase was most marked in high-grade gliomas. Our study confirmed the significant increases in Cho/NAA and Cho/tCr. Five of the seven patients we studied had grade II gliomas, thus skewing our results towards the metabolic changes of grade II gliomas. Based predominantly on patients with grade II gliomas, our results are consistent with the findings in the 3T MRS literature and the study by Li et al. They also observed that the NAA/tCr ratio was significantly decreased for both grade II and grade III gliomas [24], a finding confirmed in our study. The ratio of mIns/tCr was found to be significantly increased for both grade II and grade III gliomas in the study by Li et al. [24]. In our study, the mins/tCr ratio was also increased in the tumour region. A later 7T MRS study by Hangel et al. also confirmed the studies of Li and co-workers while using water but not tCr as references [25]. Notably, the increase in the concentration of mIns was not significant, but when compared to a non-significant decrease in the concentration of tCr, the pattern was still the same.

Glutamine (Gln) is the precursor of glutamate (Glu). Neurons turn glutamine into glutamate, which they use as an excitatory neurotransmitter. Astrocytes, on the other hand, turn glutamate into glutamine to avoid glutamate-induced excitotoxicity, which will lead to neuronal apoptosis. This is known as the glutamine–glutamate cycle [15]. Gln and Glu spectra are visible but not separable at 3T, while both are visible and separable at 7T. Li et al. and Hangel et al. found that Gln/tCr was increased for both grade II and grade III [24,25]. In our study, Gln/tCr was not significantly increased, when compared to the praecuneus in the individual patient nor when compared to the healthy controls. The limited number of subjects might have caused an increase to be non-significant. Furthermore, Li et al. found that Glu/tCr was significantly reduced in grade II but not in grade III [24]. In contrast, Hangel et al. observed a decrease in both grade II and III gliomas [25]. Our study did not allow us to differentiate as there were only two grade III patients. It should be noted that Glu is not just a neurotransmitter but is also a metabolite in the TCA cycle. Thus, changes in Glu could be caused by a simple reduction in neurons or due to the Warburg effect, with glycolysis with lactate production in the tumour region. The fact that the reduction in Glu was more marked in grade II gliomas would, in our opinion, indicate a reduced number of neurons as the primary cause of the Glu reduction.

In the present study, patient no. 5 had the lowest concentration of 2-HG (0.144 mM). The 2HG CRLB was 199%, which was above the common rejection threshold of 50% [45]. Moreover, the fit of the spectrum for the patient did not suffer when 2-HG was excluded from the basis set, unlike the other patients’ scans. Despite this, the histological analyses of the resected tissue showed the IDH mutation. Previous studies showed that intratumorally, 2-HG levels correlate with the cellular density of mutant IDH1 glioma tumours in which low tumour cellularity will lead to limited 2HG-MRS sensitivity for the detection of low 2-HG levels [46]. As stated earlier, patient no. 6 had not undergone surgery nor stereotactic biopsy. Patient no. 6 had the highest concentration (1.030 mM) of the examined patients. To use this analysis to determine whether or not the mutation is present would be premature, but the acquired data point in that direction. Emir et al. [19] found that only tumours exhibiting IDH mutations showed a 2-HG signal, with a cut-off CRLB of 30%. Berrington et al. [47] studied glioma patients, seven of whom had the IDH mutation. All seven showed the 2-HG signal with a CRLB of 9% or less. Aside from patient no. 5, our findings correspond with the two above-mentioned studies.

It is known from 3T MRS that lactate is increased in gliomas with necrosis, which is a feature in high-grade gliomas [10]. In our study, the lactate/tCR ratio was not significantly different in tumour tissue compared to the praecuneus in either patients or controls, although a trend towards an increase was seen. In the recruitment process, we only included patients who had suspected low-grade gliomas based on radiological findings on a 3T MRI, which meant no patients with visible contrast enhancement, necrosis, oedema, or bleeding. Despite this, the histological classification was grade III for two of the patients, with only patient 5 displaying any sign (at a very early stage) of necrosis on the structural scans. This may therefore explain the lack of significant differences between the tumour and praecuneus in both groups.

### 4.4. Perspective for the Future—3T and 7T MRS in the Clinical Setting

A decrease in the neuronal marker NAA appears to be a main finding in gliomas in both 3T and 7T MRS studies. Cho, a marker of cell proliferation, would expectedly be increased in the growing tumour tissue. This leads to a special interest in the NAA/Cho ratio as well as in the NAA/tCr and Cho/tCr ratios as in vivo biomarkers for tumours growing in the brain. They have some still-limited value in grading the tumour pathology. Including mIns, Gln, and Glu in the biochemical profile provides further possibilities for the characterization of a tumour’s pathology, its grade, and the treatment response. In the clinical setting, the use of MRS in the evaluation and treatment of patients has, at present, not gained wide use but remains as a valuable tool for future clinical development.

### 4.5. s-LASER or MEGA s-LASER

J-difference editing sequences, such as MEGA-sLASER, are preferred for the detection of low concentrations of metabolites (e.g., GABA or oncometabolite cystathionine) which overlap with more abundant metabolites [48,49]. They can also be utilised specifically for resolving the 2HG signal at 4.02 ppm, which is sufficiently distant from the co-edited resonances of overlapping metabolites [29]. However, this technique might be more sensitive to B_0_ and B_1_ inhomogeneities and 2-HG resonance at 4.02 ppm, which is near the water signal and may be obscured by the water signal due to reduced editing and water-suppression efficiency [29]. Therefore, the choice of either sLASER or MEGA-sLASER in a clinical setting depends on the biomarkers of interest.

### 4.6. Limitations

Despite replicating findings in the 3T and 7T literature, our exploratory study was naturally limited by a low sample size. This lowers the sensitivity during statistical group comparisons, i.e., there may be differences compared to the controls and the patients’ own praecuneus regions that were not “captured” in our limited patient population. Such sample sizes also require a cautious interpretation of the results and findings in general since we do not have the power to conclude likely relationships when we do not know if our patients are representative of a larger population with the same mix of low- versus high-grade gliomas. Compared to MRSI studies, a single-voxel MRS study also only has as many control regions as time allows. The difference between a tumour and a control region in patients might have been significant if the control voxel had been placed somewhere other than in the praecuneus. This is the reason behind MRSI, which allows for the evaluation of peritumoural and distant regions and thus poses an inherent limitation in single-voxel MRS.

Another limitation is the selection of the reference region, which in our case, was the praecuneus. Due to the difference in metabolites in different regions of the brain, it might have been better to choose the contralateral region. Some of the tumours were situated proximal to the midline, which means it it would not have been possible to use the contralateral region as a reference in the patients without having an overlap with the tumour region.

Lastly, the mentioned lack of systematic QA overview studies for 7T MRS data from gliomas (see Section 3.2) may present a bias for small-sampled 7T MRS/MRSI studies in which SNR and FWHM thresholds are used.

Finally, we would like to mention that a study has compared 3T and 7T MRS in patients with gliomas. At first glance, the higher signal-to-noise ratio obtained with 7T when compared to 3T should be considered advantageous as it allows for a higher resolution and/or shorter scan time as well as for the separation of more substances. Still, the pro and contra appear to be more complicated. Thus, the spatial coverage was found to be reduced at 7T, and the best contrast in the Cho/NAA ratio between a normal-appearing brain and a tumour may be obtained at 3T [50].

### 4.7. Concluding Remarks

In this exploratory study, we observed metabolic changes in brain gliomas that were consistent with the existing 3T and 7T MRS/MRSI literature. Although limited by our low sample size, differences between tumour regions and normal brain regions in controls from a previous 7T MRSI study [24] were replicated in the present research report. This indicates the feasibility of performing single-voxel MRS measurements when metabolite concentrations in the tumour are the only targeted biomarkers, although the desire to evaluate peritumoral regions would require more voxels and likely make MRSI the superior choice. Nevertheless, our findings with single-voxel MRS open up the possibility of adding other clinical/research sequences of interest while keeping an acceptable scan duration in terms of patient tolerability at 7T MRI. Special attention may be directed towards the IDH mutation as 2-HG is the most time-consuming MR metabolite to study and as this mutation predicts a more favourable outcome with longer survival.

## Figures and Tables

**Table 1:** Information regarding the patients.

Pt. No.	1	2	3	4	5	6	7
Sex	M	F	M	M	M	F	M
Age	29	34	44	58	41	27	36
WHO Grade	II	III	II	II	III	II *	II
Type of glioma	Astrocytoma	Astrocytoma	Oligodendroglioma	Astrocytoma	Oligoastrocytoma	Unknown	Astrocytoma
IDH—mutation	IDH-1	IDH-1	IDH-1	IDH-1	IDH-1	Unknown	IDH-1
2-HG (mM)				0.206	0.144	1.03	0.833
CRLB 2-HG				40%	199%	19%	17%
FWHM (Hz)				7.748	6.854	5.96	5.96
S/N-ratio				17	10	29	30
MGMT	Methylated	Methylated	Methylated	Methylated	Methylated	Unknown	Methylated
Tumour location	L. Occipital	L. Temporal	R. Frontoparietal	L. Parietal	L. Insula	L. Frontal	L. Frontal
Clinical presentation	Migraine auras	Visual symptoms after head trauma	Visual symptoms; suspicion of TIA	Left occipital infarction	Epileptic seizure	Focal neurological deficit	Tinnitus
Distance to Praecuneus	0.9 cm	9 cm	0 cm, situated laterally to the praecuneus	Parietally overlapping the praecuneus	4.3 cm	0 cm	3 cm

* The diagnosis of patient 6 was based only on radiological findings.

**Table 2:** Median ratio, mean ratio, and standard deviation (SD) for the different metabolites in mmol/L.

	Tumour (Median, Mean + SD) (Mean + SD)	Praecuneus, Tumour Patient (Median, Mean + SD) (Mean + SD)	Praecuneus, Healthy Control (Median, Mean + SD) (Mean + SD)	Tumour vs. Praecuneus in Patients (*p*-Values)	Tumour vs. Praecuneus in Controls (*p*-Values)	Praecuneus in Patients vs. Controls (*p*-Values)	Coefficient of Variation in Controls
**tCr** CRLB	3.30, 3.55 ± 1.21 3.57 ± 2.56	5.98, 5.71 ± 0.44 2.14 ± 0.35	5.89, 5.90 ± 0.48 2.14 ± 0.35	*p* = 2.3·× 10^−3^	* *p* = 1.2·× 10^−3^	*p* = 0.46	8%
**Cho** CRLB	1.44, 1.38 ± 0.38 5.43 ± 4.63	0.92, 0.84 ± 0.27 10.1 ± 12.2	0.94, 0.87 ± 0.23 13.6 ± 10.8	*p* = 0.05	*p* = 0.05	*p* = 1.00	26%
**NAA** CRLB	1.53, 2.08 ± 1.08 5.71 ± 2.76	8.34, 7.99 ± 0.87 2.29 ± 2.55	8.17, 8.05 ± 0.77 2.14 ± 0.35	* *p* = 5.8·× 10^−4^	* *p* = 5.8·× 10^−4^	*p* = 1.00	10%
**mIns** CRLB	4.61, 4.05 ± 1.23 5.14 ± 2.36	3.91, 3.91 ± 0.51 5.71 ± 2.05	3.89, 3.76 ± 0.59 5.71 ± 2.25	*p* = 0.46	*p* = 0.38	*p* = 0.71	16%
**Lactate** CRLB	1.12, 1.65 ± 1.16 17.9 ± 12.7	0.43, 0.38 ± 0.29 339 ± 418	0.39, 0.36 ± 0.21 214 ± 324	*p* = 0.01	*p* = 4.1·× 10^−3^	*p* = 0.90	59%
**GABA** CRLB	0.54, 0.56 ± 0.21 38.7 ± 8.33	1.55, 1.70 ± 0.45 19.4 ± 5.29	1.33, 1.48 ± 0.36 19.6 ± 4.10	* *p* = 5.8·× 10^−4^	* *p* = 5.8·× 10^−4^	*p* = 0.46	24%
**Gln** CRLB	1.69, 1.74 ± 0.64 14.6 ± 5.68	2.22, 2.16 ± 0.35 13.4 ± 4.72	1.95, 2.00 ± 0.31 12.9 ± 1.46	*p* = 0.26	*p* = 0.21	*p* = 0.32	16%
**Glu** CRLB	1.42, 1.51 ± 1.07 25.6 ± 22.4	6.87, 6.75 ± 0.74 3.43 ± 0.50	6.95, 7.02 ± 0.43 3.14 ± 0.35	*p* = 5.8·× 10^−4^	* *p* = 5.8·× 10^−4^	*p* = 0.62	6%
**Cho/tCr**	0.39, 0.41 ± 0.09	0.16, 0.15 ± 0.05	0.15, 0.15 ± 0.05	* *p* = 5.8·× 10^−4^	* *p* = 5.8·× 10^−4^	*p* = 0.38	30%
**Cho/NAA**	0.84, 0.79 ± 0.34	0.11, 0.10 ± 0.04	0.11, 0.11 ± 0.04	* *p* = 5.8·× 10^−4^	* *p* = 5.8·× 10^−4^	*p* = 0.90	33%
**NAA/tCr**	0.58, 0.58 ± 0.17	1.41, 1.40 ± 0.09	1.34, 1.37 ± 0.09	* *p* = 5.8·× 10^−4^	* *p* = 5.8·× 10^−4^	*p* = 0.46	7%
**mIns/tCr**	1.10, 1.18 ± 0.30	0.70, 0.68 ± 0.06	0.64, 0.63 ± 0.06	* *p* = 5.8·× 10^−4^	* *p* = 5.8·× 10^−4^	*p* = 0.13	10%
**Lactate/tCr**	0.47, 0.52 ± 0.36	0.08, 0.07 ± 0.06	0.06, 0.06 ± 0.04	*p* = 2.3·× 10^−3^	*p* = 2.3·× 10^−3^	*p* = 0.90	57%
**GABA/tCr**	0.13, 0.17 ± 0.07	0.26, 0.30 ± 0.08	0.25, 0.25 ± 0.06	*p* = 0.04	*p* = 0.07	*p* = 0.38	23%
**Gln/tCr**	0.43, 0.52 ± 0.19	0.36, 0.38 ± 0.07	0.34, 0.34 ± 0.07	*p* = 0.32	*p* = 0.038	*p* = 0.46	21%
**Glu/tCr**	0.51, 0.43 ± 0.24	1.16, 1.18 ± 0.10	1.20, 1.20 ± 0.14	* *p* = 5.8·× 10^−4^	* *p* = 5.8·× 10^−4^	*p* = 0.71	11%
**Glu/Gln**	0.63, 0.89 ± 0.61	2.95, 3.24 ± 0.80	3.37, 3.60 ± 0.63	* *p* = 5.8·× 10^−4^	* *p* = 5.8·× 10^−4^	*p* = 0.26	17%
**Lipid (0.9 ppm)** CRLB	0.39, 0.42 ± 0.43 312 ± 469	0.66, 0.87 ± 0.75 35.3 ± 6.92	0.43, 0.46 ± 0.16 43.3 ± 8.2	*p* = 0.25	*p* = 0.64	*p* = 0.44	
**Lipid (1.3 ppm)** CRLB	1.44, 1.28 ± 1.36 443 ± 520	1,01, 1.48 ± 1.99 327 ± 461	0.73, 0.87 ± 0.59 193 ± 356	*p* = 0.9	*p* = 1.0	*p* = 1.0	
**Lipid (2.0 ppm)** CRLB	0.21, 0.33 ± 0.41 351 ± 456	0.32, 0.32 ± 0.29 216 ± 352	0.13, 0.22 ± 0.17 103 ± 73	*p* = 0.9	*p* = 1.0	*p* = 0.54	
**FWHM (Hz)**	10.13 ± 7.15	8.64 ± 1.49	7.75 ± 0.89	*p* = 0.622	*p* = 0.434	*p* = 0.236	
**SNR**	24.43 ± 13.61	38.29 ± 9.94	46.86 ± 5.46	*p* = 0.07	*p* = 0.004	*p* = 0.093	

Median ratio, mean ratio and standard deviation (SD) for the different metabolites in mmol/l. CRLB are in percentage. We used a Bonferroni-corrected alpha of 0.00147 due to multiple comparisons. We studied 17 metabolic ratios twice; thus, our alpha level is equal to 0.05/(17 × 2). Significant *p*-values are marked with an asterisk (*).

**Table 3:** Shows the FWHM and SNR for the respective spectra.

Spectral Quality	Voxel Placement	FWHM (Hz)	SNR
Patient 1	Tumour	9.834	26
	Praecuneus	9.834	49
Patient 2	Tumour	27.416	6
	Praecuneus	7.748	47
Patient 3	Tumour	7.748	28
	Praecuneus	9.834	48
Patient 4	Tumour	5.96	20
	Praecuneus	9.834	28
Patient 5	Tumour	7.748	7
	Praecuneus	9.834	42
Patient 6	Tumour	5.96	42
	Praecuneus	7.748	23
Patient 7	Tumour	5.96	42
	Praecuneus	5.96	31
Healthy control 1	Praecuneus	7.748	46
Healthy control 2	Praecuneus	5.96	50
Healthy control 3	Praecuneus	9.834	42
Healthy control 4	Praecuneus	7.748	41
Healthy control 5	Praecuneus	7.748	58
Healthy control 6	Praecuneus	7.748	43
Healthy control 7	Praecuneus	7.748	48

**Table 4:** Comparison of different studies comparing metabolic ratios in gliomas to “normal” brain tissue.

	tCr	NAA	Cho	mIns	Lac	Gln	Glu	Cho/ NAA	Cho/ tCr	NAA/ tCr	mIns/ tCr	Gln/ tCr	Glu/ tCr	GABA/ tCr
Galanaud (1.5T) ^§^	−	↓	↑	↑										
Tong (1.5T)	↓	↓	(↓)					↑		↓				
Bulik (1.5 and 3T) ^#^	↓	↓	↑	↑ †	↑ ‡			↑	↑					
Caivano (3T)								↑	↑	↓				
Hangel (7T)	(↓)	↓	↑	(↑)		↑	↓							
Li (7T)								↑†	↑†	↓	↑	↑	↓	−
Our 7T Study	↓ *	↓	(↑)	−	(↑)	−	↓	↑	↑	↓	↑	−	↓	(↓)

^§^ Compared to sum of metabolites. ^#^ General statement based on review. * Only significant for tumour vs. praecuneus in healthy controls. † Only significant for grade II gliomas. ‡ Only significant for grade III gliomas. In parenthesis, trends towards.

## Data Availability

Data can be obtained by contacting the authors. The access to and use of the data must be in accordance with the rules of the Danish legislation and must be approved according to the Danish Data Protection Agency’s rules.

## Abbreviations

2-HG	2-hydroxyglutarate
Cho	Choline
CRLB	Cramer-Rao Lower Bounds
CV	Coefficient of Variation
FWHM	Full Width at Half Maximum
GABA	Gamma-Aminobutyric acid
Gln	Glutamine
Glu	Glutamate
Glx	Glutamate + Glutamine
GSH	Glutathione
IDH1/2	Isocitrate-dehydrogenase 1/2
LGG	Low-Grade Glioma
mIns	Myo-inositol
MRS	Magnetic resonance spectroscopy
NAA	N-acetylaspartate
NAAG	*N*-acetylaspartyl glutamate
sLASER	Semi-localization by adiabatic-selective refocusing
SNR	Signal-to-Noise Ratio
TCA	Tricarboxylic Acid Cycle
tCr	Total Creatine
TE	Echo time
TI	Inversion time
TR	Repetition time
VAPOR	Variable power RF Pulses with Optimized Relaxation delays
VESPA	Versatile Simulation, Pulses and Analysis
